# Surgery vs. radiotherapy in localized prostate cancer. Which is best?

**DOI:** 10.1186/1748-717X-3-23

**Published:** 2008-09-07

**Authors:** Stefan Welz, Maximilian Nyazi, Claus Belka, Ute Ganswindt

**Affiliations:** 1 Department of Radiation Oncology, Eberhard-Karls-University, Hoppe-Seyler-Str. 3, 72076 Tübingen, Germany; 2 Department of Radiation Oncology, Ludwig-Maximilians-University, Marchionini-Str. 15, 81377 Munich, Germany

## Abstract

Surgery and radiotherapy are currently accepted alternatives for the treatment of localized prostate cancer. In the absence of relevant randomized trials no decision regarding the superiority of any of the given approaches can be made. Up to now several cohort-based approaches indicate similar outcomes for both treatments. Based on a new population based approach, Merglen and co-workers recently concluded that surgery would offer the best chance of long-term control in terms of 10-year survival for T1–T3 prostate cancer patients. Unfortunately the strength of this trial is limited by several shortcomings. Most importantly, issues of radiation dosage have not been taken into account. In addition, several relevant parameters including Gleason score and PSA are not well balanced between the arms and the assignment to arbitrary risk groups does not reflect the real biological behaviour. Thus, the data provided do not support the strong conclusion issued by the authors. Based on the data available, surgery and radiotherapy still have to be considered as equally effective.

## Commentary

Despite an intense debate over the last decades, there is no agreement today on the treatment of choice for localized prostate cancer. This is mainly due to the absence of large randomized trials comparing radical prostatectomy and radiation-based approaches. Therefore, clinicians have to rely on retrospective and population-based trials which, due to their nature, comprise a considerable risk of misinterpretation and must be analyzed very carefully [1].

In this regard, a Swiss group performed a population based analysis on treatment outcome after radiotherapy or surgery for prostate cancer in the Geneva region [2], which is, unfortunately, subject to an array of methodological pitfalls and misinterpretations.

This trial is a retrospective observational cohort study on 844 T1–3 prostate cancer patients documented in the Geneva Cancer Registry with a treatment period from 1989 to 1998.

The authors state, that the 5-year overall- and cancer-specific survival rates were almost identical for radiotherapy and prostatectomy but worse for the other treatment options. After ten years, the authors noted, that prostate cancer specific survival was inferior in radiotherapy-only patients when compared to surgery-only patients. No such difference was seen when surgery-only was compared to radiotherapy plus hormonal ablation. Watchful waiting and hormonal treatment only were found to have inferior outcomes.

These conclusions are based on the endpoint "mortality from prostate cancer". Yet, the authors fail to provide a definition for this endpoint, which itself is difficult to assess. It has been shown, that the cause of death in patients with prostate cancer is easily misattributed [3]. Since this aspect, concerning the most important endpoint of the study, is totally ignored by Merglen et al, misinterpretation of the data is due to happen.

Additionally, there are several further shortcomings that limit the value of the current study, most of which concern confounding variables:

First, the Gleason score is missing in 15% of the radiotherapy patients and in only 3% of the surgical patients. Furthermore, the grouping of patients was not based on the Gleason score only, but on a mixture of Gleason score and grading. The result is, that patients with a Gleason of 7 are analyzed in the same group as patients with a Gleason of 10, despite the fact that these scores represent distinct risk populations [4]. Another important predictor for the outcome after treatment is the initial PSA level. Again, there is a severe imbalance with 37% of the radiotherapy patients having PSA levels higher than 20 ng/ml whereas only 27% of the surgical patients had such PSA values. Moreover, there was a difference of 8% in patients with a PSA level above 30 ng/ml in favour of the surgical group. Next, there is the issue of the lymph node status: There is no hint, on how patients in the radiotherapy group have been staged. Resected patients are prone to have had pathological staging, whereas radiotherapy patients probably had computed tomography, which is known to be unreliable in this regard [5,6]. Thus, another bias is introduced.

The authors employed a Cox-regression model to adjust for given imbalances. This model should be able to control for imbalanced confounding factors if the influence of these factors is equally distributed in all subgroups. Although the used Cox model is an adequate statistical tool, it is doubtful whether statistical tools may be of any use to make up for inadequate grouping of risk factors (Gleason) or for a possible systematic clinical understaging of certain patient collectives (N-status in radiotherapy patients). At no point in their paper the authors indicated possible weaknesses of this approach.

Last but not least, the authors completely neglected the importance of adequate dosage for the outcome of radiation-based treatment approaches [7-9]. In the last 15 years, advances in technical equipment allowed an escalation of the prescribed dose to the level which is standard nowadays. Since the trial by Merglen et al. was done in the late eighties, it is likely that non-optimal radiation doses were applied. Yet, the authors do not provide any data on this aspect. Considering, that study patients with radiotherapy and hormonal ablation show a comparable outcome to surgical patients, the reason could be, that hormonal therapy at least partially compensated for underdosage [10].

In conclusion, the statement issued by Merglen and co-workers that "surgery offers the best chance of long-term prostate-cancer-specific survival in particular for younger patients and patients with poorly differentiated tumours" is not supported by the data presented in their paper. Most of the obvious problems including the effects of radiation dose, clinical vs. pathological staging as well as the attribution of causes of death are not discussed by the authors. The same is true for critical aspects on the discussed issue in the papers that were already mentioned. Additionally, there are data indicating an equivalence of radical prostatectomy and radiotherapy, provided, that an adequate radiation dose is prescribed [11-16], a fact, which is neglected in the discussion.

Thus, despite the fact that observational studies are necessary to evaluate the efficacy of surgery vs. radiotherapy in prostate cancer, the paper by Merglen et al. does not add valid information. In addition, we would be highly reluctant to use these data for patient counselling as suggested by the authors "Until clinical trials provide conclusive evidence, physicians and patients should be informed of these results and their limitations".

## Competing interests

The authors declare that they have no competing interests.

## Authors' contributions

SW and UG did the literature research and drafted the manuscript, UG and MN revised the manuscript, CB and UG conceived the article and revised it. All authors read and approved the final manuscript.
